# Quercetin–Resveratrol Combination for Prostate Cancer Management in TRAMP Mice

**DOI:** 10.3390/cancers12082141

**Published:** 2020-08-02

**Authors:** Chandra K. Singh, Gagan Chhabra, Mary A. Ndiaye, Imtiaz A. Siddiqui, Jennifer E. Panackal, Charlotte A. Mintie, Nihal Ahmad

**Affiliations:** 1 Department of Dermatology, University of Wisconsin, 1300 University Avenue, Madison, WI 53706, USA; csingh@dermatology.wisc.edu (C.K.S.); gchhabra@dermatology.wisc.edu (G.C.); mndiaye@dermatology.wisc.edu (M.A.N.); imtiaz.siddiqui@cuanschutz.edu (I.A.S.); panackal@wisc.edu (J.E.P.); mintie@wisc.edu (C.A.M.); 2 William S. Middleton VA Medical Center, Madison, WI 53705, USA

**Keywords:** prostate cancer, chemoprevention, grape antioxidants, quercetin, resveratrol

## Abstract

Prostate Cancer (PCa) is a leading cause of cancer-related morbidity and mortality in men. Therefore, novel mechanistically-driven approaches are needed for PCa management. Here, we determined the effects of grape antioxidants quercetin and/or resveratrol (60 and 600 mg/kg, respectively, in diet) against PCa in Transgenic Adenocarcinoma of Mouse Prostate (TRAMP)-model in prevention and intervention settings. We found resveratrol alone and in combination significantly inhibited prostate tumorigenesis in prevention setting, while the same was seen only in combination after intervention. The observed effects were associated with marked inhibition in proliferation, oxidative stress, and tumor survival markers, and induced apoptosis markers. Utilizing PCa PCR array analysis with prevention tumor tissues, we identified that quercetin–resveratrol modulates genes involved in promoter methylation, cell cycle, apoptosis, fatty acid metabolism, transcription factors, androgen response, PI3K/AKT and PTEN signaling. Ingenuity Pathway Analysis (IPA) identified IGF1 and BCL2 as central players in two gene networks. Functional annotation predicted increased apoptosis and inhibited cell viability/proliferation, hyperplasia, vasculogenesis, and angiogenesis with dual treatment. Furthermore, IPA predicted upstream inhibition of major PCa signaling VEGF, Ca^2+^, PI3K, CSF2, PTH). Based on PCR array, we identified decreased levels of EGFR, EGR3, and IL6, and increased levels of IGFBP7 and NKX3.1, overall supporting anti-PCa effects of quercetin–resveratrol.

## 1. Introduction

Prostate cancer (PCa) is the most frequently diagnosed cancer accounting for more than one in five new cancer diagnoses affecting men in the US [1]. PCa is generally slow-growing and follows a distinct progression pattern which makes it ideal for both prevention and intervention studies [2]. Even though therapies like surgery, endocrine therapy, or radiotherapy provide control over PCa progression, a considerable number of patients ultimately advance to a metastatic, hormone-refractory state. Therefore, the identification of novel mechanism-based approaches is desired for the prevention and/or treatment of PCa. 

Historically, plant-based and dietary agents have been used medicinally in a variety of health conditions, including PCa [3,4]. The grape antioxidant resveratrol (chemically: 3,5,4′-trihydroxystilbene) is one such agent that is being extensively studied for its health-promoting effects. Resveratrol has been found to afford chemopreventive as well as therapeutic effects against PCa and other cancers [5,6,7]. Studies show that resveratrol inhibits key signaling pathways associated with tumor initiation, promotion, and progression, and modulates several pathways related to hallmark features of cancers [8,9]. The existing literature suggests that resveratrol may be useful when administered in combination with other drugs or natural agents for cancer management, including PCa [10,11]. Previously in our lab, we demonstrated that (i) resveratrol possesses pro-apoptotic effects against human PCa cells without affecting the normal prostate epithelial cells, and (ii) the anti-proliferative effects of resveratrol against PCa cells may be mediated via modulation of phosphatidylinositol 3’-kinase (PI3K)/AKT pathway and BCL2 family of proteins [12]. Additionally, in several in vitro and in vivo studies, resveratrol was shown to have promising anti-PCa effects [5,6,7]. Harper and colleagues have demonstrated that resveratrol suppresses PCa progression in Transgenic Adenocarcinoma of Mouse Prostate (TRAMP)-model [13]. Seeni et al. found that resveratrol suppresses PCa growth and induces apoptosis in Transgenic Rat for Adenocarcinoma of Prostate (TRAP) model [14]. These studies suggest that resveratrol could be developed as an effective agent for the prevention and/or treatment of PCa. However, an important issue associated with the potential clinical use of resveratrol is its low in vivo bioavailability due to its rapid metabolism (via glucuronidation, sulfation and hydroxylation) [15]. 

Quercetin (3,3′,4′,5,7-pentahydroxyflavone), another plant flavonoid, is a powerful antioxidant that may play a key role in the prevention and intervention of cancer development [16]. Apart from its antioxidant activity, quercetin also exerts an apoptotic effect on tumor cells and can block cancer progression [17]. The effects of quercetin have been demonstrated in both in vitro and animal models while exhibiting antiproliferative effects exclusively on cancerous cells [18]. Interestingly, both quercetin and resveratrol are present in red grapes, red wine and several other plants in which they have a natural association [16,19]. Like resveratrol, quercetin has also demonstrated the ability to inhibit PCa in several in vitro and in vivo studies [20,21,22,23]. Additionally, quercetin has been shown to inhibit in vivo sulfation of resveratrol, potentially leading to increased resveratrol bioavailability in vivo [24]. Therefore, quercetin–resveratrol combination may impart superior therapeutic efficacy against PCa.

Herein, we investigated the effects of dietary supplementation of quercetin in combination with resveratrol in a mouse model of PCa that spontaneously develops prostate tumors in a progression that mimics human PCa. The rationale of the study was based on the fact that (i) both polyphenols (quercetin and resveratrol) are naturally present in certain plants/plant products, (ii) quercetin is known to improve the bioavailability of resveratrol by inhibiting its sulfation, and (iii) separately, both agents have shown potential for the management of PCa in previously published in vitro and in vivo studies. Additionally, chemotherapeutic drugs in combination are generally better tolerated and more effective because combinative approaches can target multiple aspects of cancer cell function, leading to improved clinical outcomes. 

## 2. Results and Discussion

### 2.1. Quercetin–Resveratrol Combination Exerted Significant Antitumor Effects against PCa

**Figure 1:**
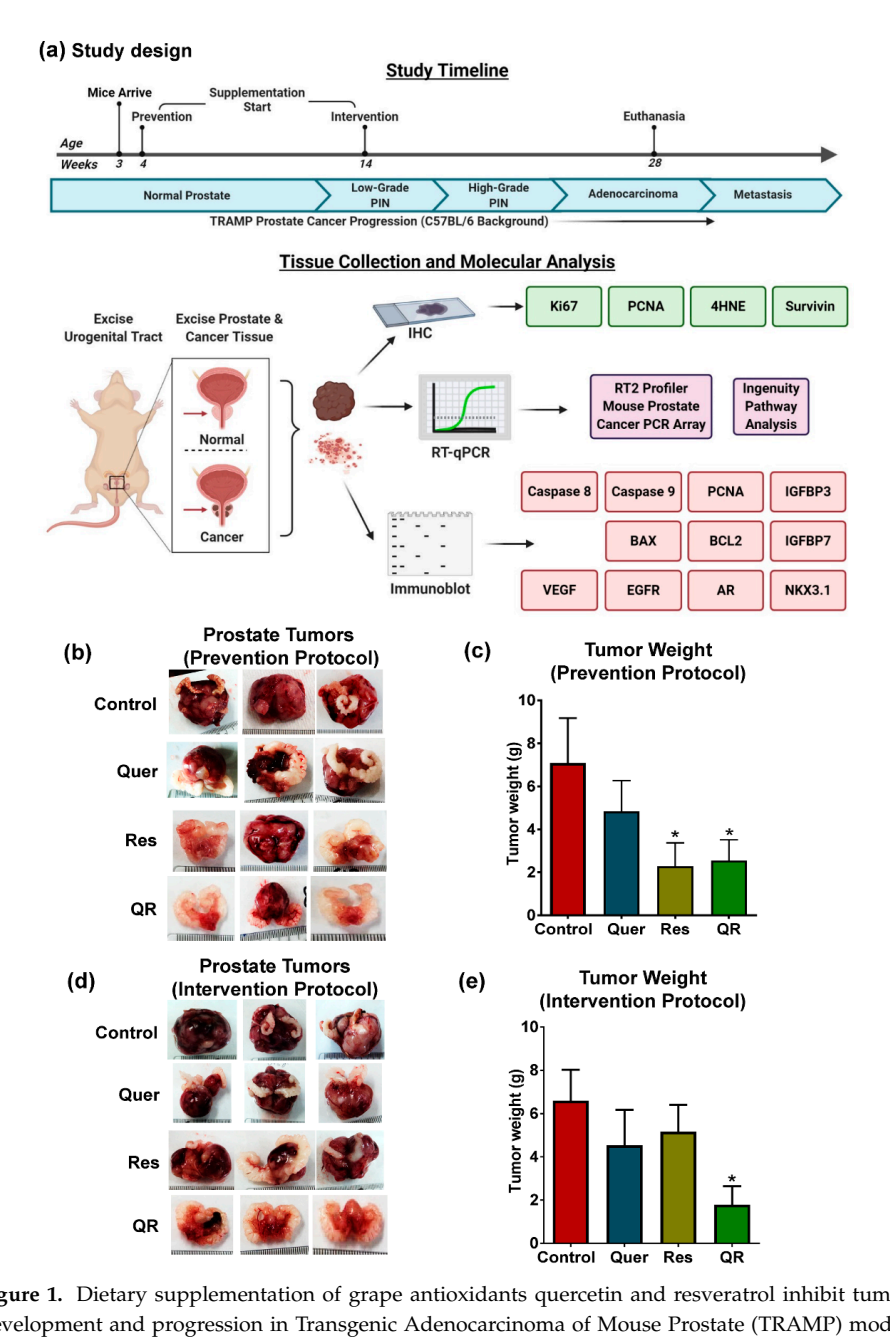
Dietary supplementation of grape antioxidants quercetin and resveratrol inhibit tumor development and progression in Transgenic Adenocarcinoma of Mouse Prostate (TRAMP) model. (**a**) Preclinical study design and the timeline for prevention and intervention settings in TRAMP mice. (**b**) Representative images of tumors with scale bar showing the size of tumors at the termination of study in prevention setting. (**c**) Graphical representation of tumor wet weight data in prevention setting. (**d**) Representative images of tumors with scale bars in the intervention setting. (**e**) Tumor wet weight data in the intervention setting. The tumor data are represented as mean ± SEM of 12 animals per group. GraphPad Prism 5 Software was used to perform statistical analyses on tumor data using one-way analysis of variance (ANOVA) followed by uncorrected Fisher’s Least Significant Difference (LSD) test (* *p* < 0.05).

Using the TRAMP mice, a clinically relevant model for PCa biology and therapeutic studies, we determined if using a combination of quercetin and resveratrol would impart better anti-cancer effects than either agent alone. These mice display the hallmark characteristics of PCa development and progression [25] and recapitulate key features of human PCa. TRAMP mice on the C57BL/6 background show epithelial hyperplasia by 8 weeks of age, progress to prostatic intraepithelial neoplasia (PIN) by 18 weeks of age, and begin to show metastases after 28 weeks of age [26,27]. Based on the characteristic features of disease progression in these mice, the preclinical study was designed as detailed in Figure 1a.

As reported earlier, TRAMP mice develop prostatic adenocarcinoma, as well as seminal vesicle epithelial–stromal (ES) tumors resembling phyllodes tumors in most of the mice [28]. Our pre-clinical data demonstrate a significant decrease in tumor size and weight in resveratrol and quercetin–resveratrol groups in the prevention setting (Figure 1b,c). In this setting, we did not observe any significant benefit with the combination as both resveratrol alone and in combination show similar antitumor effects. Interestingly, in the intervention setting where supplementation was started after the disease was already established, a significant decrease in tumor size and weight was noticed only in quercetin–resveratrol treatment group (Figure 1d,e). This is a promising observation, as most agents fail to achieve therapeutic efficacy with the advancement of the disease. This suggests the possibility of enhanced or differentially expressed genes/proteins that quercetin–resveratrol can target after the onset of disease. This may also explain why we see no additive effect with the prevention protocol, as these enhanced targets may be unavailable before the development of PIN.

Over the course of the study, ~25% of mice within the control groups did not develop tumors. It is possible that similar percentages of mice in the treatments did not develop tumors as well. Although male TRAMP mice are traditionally thought to develop prostate tumors in a uniform fashion [29], strain backgrounds have been shown to influence transgene expression levels and phenotypic penetrance of PCa in TRAMP mice [30]. The tumor weight data presented in Figure 1c,e include all mice in the cohorts, as excluding mice which failed to develop tumors did not affect the results of this study other than reducing the somewhat large error bars (Figure S1).

During the study, no unexpected changes in the body weight of any mice were noticed. At the time of euthanasia, animals were examined and it was determined that there were no gross organ abnormalities, indicating no adverse effects on the parameters evaluated. We found that the quercetin and resveratrol doses used in this study were well tolerated and there were no significant differences in consumption of any of the diets. We planned the doses of quercetin and resveratrol to be used in this study so that when administered in diet, the mice would receive 10 and 100 mg/kg b. wt. per day of quercetin and resveratrol, respectively. These dose calculations were based on the expected average dietary intake for a 30 g mouse being ~5 g. However, the actual consumption of diet/day was ~4 g during this study. With this difference, the actual doses of quercetin and resveratrol were 8 and 80 mg/kg b.wt per day, respectively. Based on the animal to human dose translation model, these correspond to human equivalent doses of 0.65 and 6.49 mg/day, respectively [31]. 

### 2.2. Anti-PCa Effect of Quercetin–Resveratrol Is Associated with Marked Inhibition in Markers of Cell Proliferation, Oxidative Stress, and Tumor Survival, as well as Induction of Apoptosis

**Figure 2:**
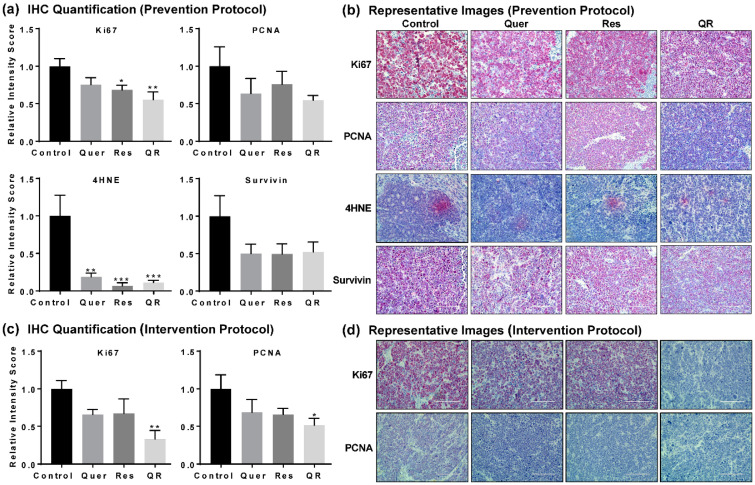
Quercetin–resveratrol supplementation inhibits markers of cell proliferation, oxidative stress, and tumor survival in prostate cancer. (**a**) Semi-quantitative immunohistochemical (IHC) analysis and (**b**) representative images of proliferative markers Ki67 and PCNA, oxidative stress biomarker 4HNE, and tumor survival marker Survivin with tumor tissues from the prevention setting. (**c**) Semi-quantitative and (**d**) representative images of IHC analysis of Ki67 and PCNA in the intervention setting. Images were obtained at 10, 20 and 40× magnification for the overall analysis of the tissue samples, however, only representative images at 40× magnification are presented here. Semi-quantitation was performed using ImageJ Fiji (version 1.2) and statistical significance was determined using GraphPad Prism 5 software using one-way ANOVA followed by uncorrected Fisher’s LSD test (* *p* < 0.05, ** *p* < 0.01, *** *p* < 0.001). The data presented are mean ± SEM of six animals per group.

Next, we performed immunohistochemical (IHC) analysis of key proteins (Ki67, proliferating cell nuclear antigen (PCNA), 4-hydroxynonenal (4HNE), and Survivin) in prostate tumors to give potential insight to the molecular mechanisms of quercetin and resveratrol. Ki67 and PCNA are biomarkers of cell proliferation, rapid growth and cellular division. 4HNE is a biomarker of oxidative stress as it is produced during the degradation of lipids in cell membranes by free radicals. Survivin is a biomarker of cell survival as it prevents programmed cell death. IHC images were semi-quantified as described elsewhere [32]. We found significant decreases in 4HNE in all three treatment groups, suggesting anti-PCa effects of quercetin and/or resveratrol may arise by reducing the oxidative damage caused by 4HNE in PCa. We also found a marked decrease in cell proliferation markers Ki67 and PCNA, and cell survival marker Survivin in response to quercetin and/or resveratrol in the prevention setting (Figure 2a,b). Interestingly, the effects were more apparent in the quercetin–resveratrol group for Ki67 and PCNA, suggesting an additive effect of combination at the molecular level. This was further supported by our data in intervention settings, where the quercetin–resveratrol group showed a significant decrease in Ki67 and PCNA (Figure 2c,d).

**Figure 3:**
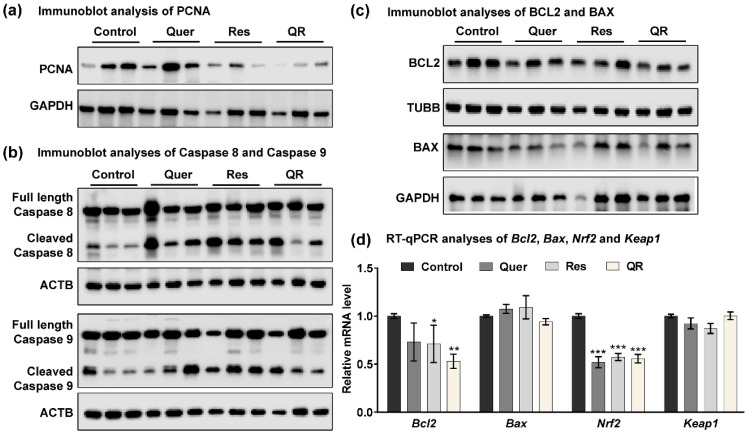
Quercetin–resveratrol supplementation induces apoptosis in prostate tumors. (**a**) Immunoblot analysis of PCNA protein. GAPDH was used as a loading control. (**b**) Immunoblot analyses of full length and cleaved caspases-8 and -9 proteins in TRAMP tumor tissues. β-actin (ACTB) was used as a loading control. (**c**) Immunoblot analyses of BCL2 and BAX proteins. β-tubulin (TUBB) and GAPDH were used as loading controls. (**d**) RT-qPCR analyses of *Bcl2*, *Bax*, *Nrf2*, and *Keap1* mRNA levels in TRAMP tumor tissues. Data are represented as mean ± SEM. A one-way ANOVA with Tukey’s multiple comparison test was performed (* *p* < 0.05, ** *p* < 0.01, *** *p* < 0.001).

For subsequent studies here, we used tumor tissues from prevention trials. We were interested in exploring the molecular differences between resveratrol and quercetin–resveratrol treatments, as similar antitumor effects between these treatments were only seen in the prevention trial. Proliferation marker PCNA is key molecule in deciding the fate of cells [33], and when present in low quantities, cells proceed to apoptosis, which is always a desirable antitumor action. We reconfirmed the modulation in PCNA in response to quercetin–resveratrol using immunoblot analysis. We found marked decrease in PCNA especially in resveratrol alone and quercetin–resveratrol group (Figure 3a), further supporting the PCNA IHC data. Next, we performed immunoblot analysis for caspases-8 and -9, as representatives of the extrinsic and intrinsic apoptosis pathways, respectively. Both pathways trigger apoptosis through the cleavage of downstream executioner proteins. Interestingly marked increases in the cleavage of both caspases-8 and -9 were noticed in all three treatment groups compared to control (Figure 3b), indicating the involvement of both extrinsic and intrinsic apoptosis pathways in the inhibition of prostate tumors. Subsequently, we assessed the effects of treatments on BCL2 and BAX proteins that regulate the mitochondrial and intrinsic apoptotic responses [34]. Using immunoblot and RT-qPCR analyses, we found a marked decrease in BCL2 in all the treatment groups with a greater decrease in the quercetin–resveratrol group (Figure 3c,d, respectively). This suggests that there is an association between the treatments and BCL2, and it may play a role in the anti-PCa effects of these antioxidants. BAX, on the other hand, showed no appreciable difference at either the protein (Figure 3c) or mRNA (Figure 3d) level. 

To follow up on the IHC results on 4HNE expression, we assessed other effectors related to oxidative stress. 4HNE has been implicated as a potential activator of nuclear factor-erythroid 2 related factor (NRF2), which interacts with antioxidant response element (ARE) to activate genes in response to oxidative stress [35]. mRNA analysis of PCa tissues identified decreased *Nrf2* levels in all three treatment groups (Figure 3d), suggesting an inhibitory effect of quercetin and resveratrol on the NRF2 signaling pathway. NRF2 is known to play an important role in cell defense and survival against endogenous/exogenous stresses, and generally, its overexpression in cancer cells enhances the expression of cytoprotective genes, resulting increased cell proliferation and inhibition of apoptosis [36]. Kelch-like ECH Associated Protein 1 (KEAP1) is known to bind NRF2 to facilitate its degradation via the proteasome. RT-qPCR analysis in tumor tissues found no changes in *Keap1* expression (Figure 3d). The result of this study is in accordance with our recently published study where NRF2 was found to be inhibited by dietary grape supplementation in a mouse model of skin cancer [19]. Our study also indicates that NRF2 is supportive of prostate tumor survival. Collectively, our data suggest that anti-PCa effects of quercetin and resveratrol were associated with modulation in key signaling related to cell proliferation, oxidative stress, tumor survival, and apoptosis.

### 2.3. Anti-PCa Effects of Quercetin–Resveratrol Are Associated with Key PCa-Related Genes

**Figure 4:**
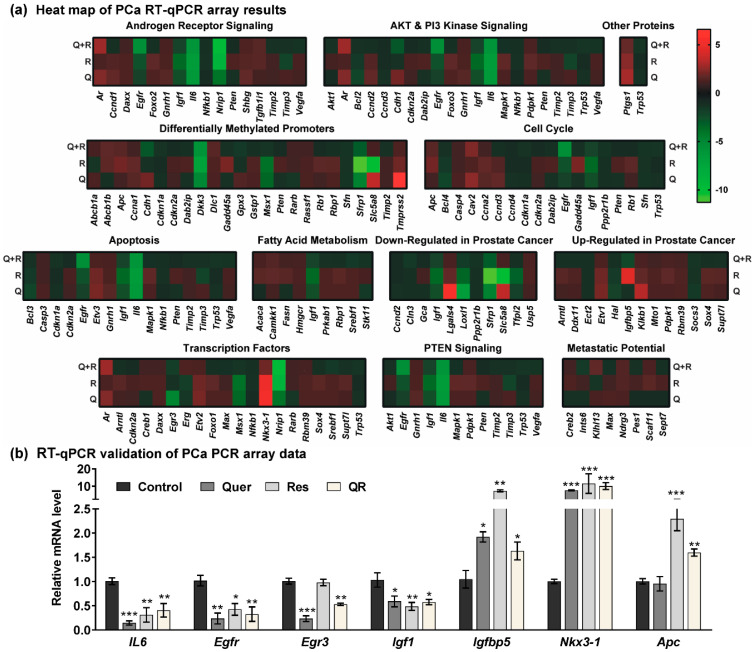
Quercetin and/or resveratrol modulate the expression of key PCa-related genes. The expression profiles of 84 genes involved in 12 different PCa-related phenomena were assessed using a Qiagen PCa RT^2^ Profiler PCR array. (**a**) Heat maps of the gene expression in quercetin (Q), resveratrol (R), and quercetin–resveratrol (QR) treated tumors are represented as the fold change compared to the control tumors. The assay was performed using two biological pools (as described in materials and methods) in triplicate within each group. Increased levels are indicated by red and decreased levels are green. (**b**) Validation of key PCa genes identified from the PCa PCR array was performed by RT-qPCR analysis. Data are presented as the mean ± SEM of two biological pools of three animals per group (*n* = 6) in technical triplicate. A one-way ANOVA with Tukey’s multiple comparison test was performed using Graphpad Prism 5 software (* *p* < 0.05, ** *p* < 0.01, *** *p* < 0.001).

In order to determine the genes/pathways associated with the observed responses of quercetin and/or resveratrol in PCa, we used a commercially available mouse PCa RT^2^ Profiler PCR array to profile the expression of 84 key PCa-related genes. The analyses of PCa-related gene expression in response to quercetin and/or resveratrol are summarized in Table S1. A heat map of PCa PCR array data show that resveratrol, quercetin, and their combination modulate several important genes related to AR, PI3K/AKT and PTEN signaling (Figure 4a). The heat map also shows the modulation in genes involved in promoter methylation, cell cycle, apoptosis, fatty acid metabolism, transcription factor, metastatic potential, and other PCa-related genes. 

Based on the analysis of the Qiagen RT^2^ Profiler PCa PCR array, we observed the modulation of several key genes known to affect the development and progression of prostate tumors. For validation of PCR array data, we selected a cut-off criteria of ≥ two-fold change in any one treatment group with statistically significant change (*p* < 0.05), and ≥ 1.5-fold change in any other group. Of 84 genes tested, 22 were identified with these parameters for further analysis (Table 1). The expression of these genes was validated using RT-qPCR analysis, and genes with significant expression after validation are shown in Figure 4b. Validation data for inconclusive or non-significant data are shown in Figure S2.

**Figure 5:**
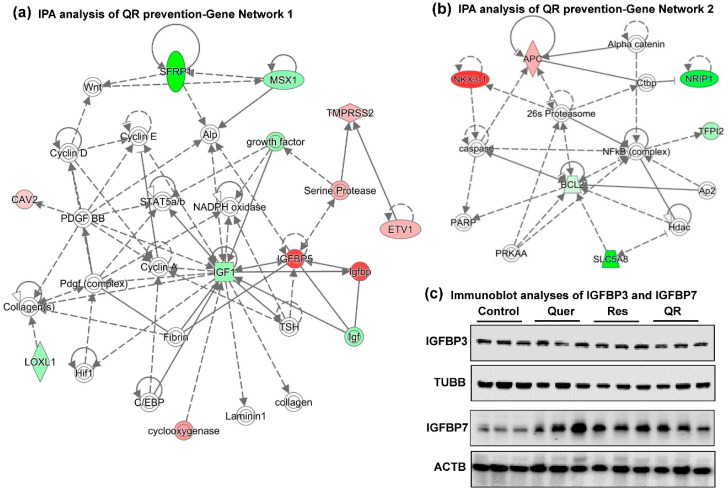
Gene networks analysis of quercetin–resveratrol modulated genes suggest IGF1 and BCL2 as key signaling nodes associated with anti-PCa effects. Differentially expressed genes from PCa PCR array with cut-off criteria of ≥2-fold change in any one treatment group with statistically significant change (*p* < 0.05), and ≥ 1.5-fold change in any other group (total 22 genes) were uploaded in Ingenuity Pathway Analysis (IPA) software. (**a**) Gene network 1 indicating IGF1 inhibition as a key player interacting with most of the genes in the network. (**b**) Gene network 2 indicating BCL2 inhibition as another important player interacting with most of the genes in the network. Red indicates upregulated genes, green indicates downregulated genes, and uncolored nodes indicate genes not included in the PCR array but appeared during IPA analysis to connect the genes of the network. Solid lines indicate robust interactions, whereas dashed lines are significant but less frequent. (**c**) Immunoblot analyses of IGFBP3 and IGFBP7 proteins. TUBB and ACTB were used as a loading control.

To explore the gene networks and pathways related to these genes, we used Ingenuity Pathway Analysis (IPA) software, which identified two gene networks (Figure 5a,b). These two networks of interacting genes show links to other crucial genes that were found during network generation (indicated with uncolored nodes), supporting the antitumor properties of dietary supplementation with quercetin–resveratrol. Exploration of gene network 1 indicates decreased IGF1 (Insulin-like growth factor) as a central regulatory player interacting with most quercetin–resveratrol modulated genes as well as several other molecules that appeared during network generation. Similarly, decreased BCL2 emerges as a gene with most interacting partners in IPA network 2. This is in accordance with our findings as validated above for *Igf1* (Figure 4b) and BCL2 (Figure 3c,d). IGF1 is one of the most potent natural activators of the AKT signaling pathway, a stimulator of cell growth and proliferation, and a potent inhibitor of apoptosis. This is an important finding, as IGF1 has been implicated in PCa development and progression. Epidemiological studies have established a link of elevated blood levels of IGF1 and risk of developing advanced PCa [37,38]. Also, IGF1 overexpression in the prostate basal epithelial layer of transgenic mice results in prostate adenocarcinoma similar to human PCa [38]. Interestingly, resveratrol has been demonstrated to decrease IGF1 levels and suppress PCa progression in TRAMP mice [13]. Quercetin treatment has also been shown to suppress IGF1 signaling in mouse skin cancer [39]. Our findings of decreased IGF1 in response to resveratrol, quercetin and combinations suggest a means of protecting the prostate by reducing the potential for androgen-independent growth often associated with the IGF1-signaling pathway.

Because the IGF1 signaling pathway has been associated with PCa progression, and Igf1 in gene network 1 interacts with Igfbp (Insulin-Like Growth Factor Binding Protein), especially *Igfbp5*, we investigated if quercetin–resveratrol could regulate key proteins in this pathway. Our PCR array experiment together with validation data demonstrated an upregulation of *Igfbp5* in resveratrol alone group only. In quercetin and quercetin–resveratrol combination groups, the results were inconclusive, as validation data did not match with PCR array data, suggesting further research is needed (Figure 4b, Table 1). IGFBP5 is known to alter the interaction of IGF with their cell surface receptors and therefore seems to control other genes in the IGF family [40]. However, IGFBP5 is also known to exert IGF-independent actions. The expression level of IGFBP5 differs context-specifically, mostly shown as a tumor suppressor although in some cancers as tumor promoter (reviewed in [41]). Androgen receptor (AR) is known to upregulate IGFBP5 in a human PCa xenograft [42]. One study also suggests that the upregulation of IGFBP5 after castration serves to enhance IGF1 bioactivity and accelerates progression to androgen independence in PCa models [43]. Our results are contrary to earlier findings in PCa and therefore need further validation to determine what is exactly happening.

Approximately 98% of IGF1 is bound to one of six binding proteins (IGFBP). IGFBP3, the most abundant protein, accounts for 80% of all IGF1 binding, which binds in a 1:1 molar ratio. Although not popular in clinical practice, PCa progression in humans is often monitored by following the serum levels of IGF1, and IGFBP3. Studies have shown that an elevated level of IGF1 with a concomitant decrease of IGFBP3 in serum is associated with elevated PCa risk. Additionally, IGFBP3 crosstalk with IGFBP7 is known to be involved in a variety of cancers, including PCa [44]. Using immunoblotting, we found no change in IGFBP3, whereas IGFBP7 was found to be upregulated in response to all treatment groups (Figure 5c). Like IGFBP5, IGFBP7 expression in cancers is ambiguous. Even in PCa, two studies show its downregulation contrary to one showing upregulation (reviewed in [44]). 

### 2.4. Functional Annotation of Quercetin–Resveratrol Modulated Genes Predicts Cumulative Antitumor Actions and Upstream Inhibition of Major PCa-Associated Pathways

**Figure 6:**
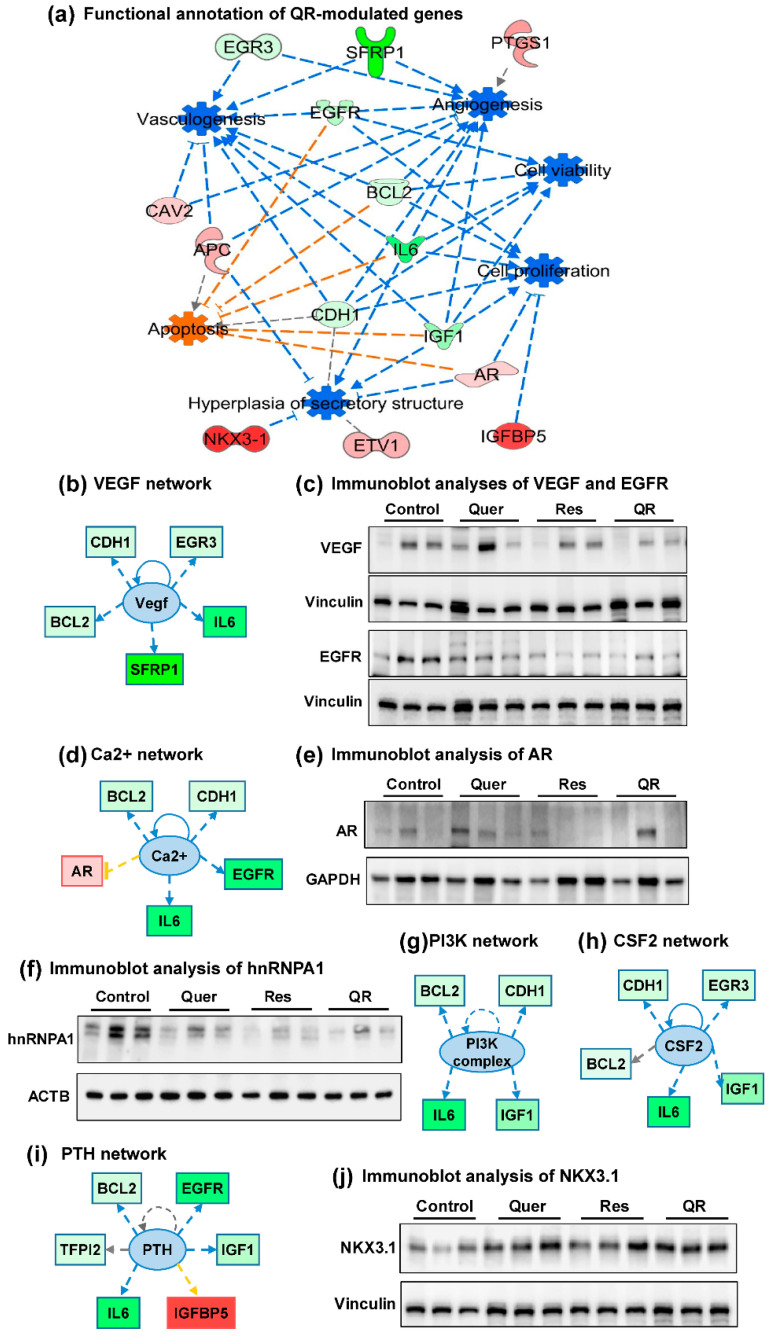
Analysis of quercetin–resveratrol (QR)-modulated genes suggest cumulative antitumor actions and upstream inhibition of major PCa signaling pathways. (**a**) Functional annotation showing cumulative actions of QR-modulated genes: increased apoptosis and inhibition of cell viability/ proliferation, hyperplasia, vasculogenesis, and angiogenesis. (**b**,**d**,**g**–**i**) Using IPA, upstream regulator analysis identified genes potentially involved in changes seen in RT-qPCR analyses. Genes from array are in red (upregulated) and green (downregulated), while predicted functions and upstream regulator genes and interaction lines are in orange (activation) and blue (inhibition). Lines in yellow indicate findings inconsistent with the state of downstream molecules. The gray dotted line shows unpredicted effects. Immunoblot analyses of (**c**) vascular endothelial growth factor (VEGF) and epidermal growth factor receptor (EGFR), (**e**) androgen receptor (AR), (**f**) hnRNPA1, and (**j**) NKX3.1 proteins. Vinculin, ACTB, and GAPDH were used as loading controls.

IPA was used to identify the cumulative actions of altered genes to understand the quercetin–resveratrol-mediated chemoprotective response against PCa. IPA analysis predicted induction of apoptosis and inhibition of cell viability/proliferation, hyperplasia, vasculogenesis and angiogenesis, in response to quercetin–resveratrol combination (Figure 6a). These findings are important because these responses are critically essential for any antitumor actions. The genes indicated in these cumulative actions are *Egr3*, *Sfrp1*, *Ptgs1*, *Egfr*, *Cav2*, *Bcl2*, *Apc*, *Il6*, *Cdh1*, *Igf1*, *Ar*, *Etv1*, *Igfbp5* and *Nkx3.1*. Individually, these genes are also known to affect the development and progression of tumors. 

IPA also predicted upstream inhibition of major PCa signaling pathways viz. vascular endothelial growth factor (VEGF), Ca^2+^, PI3K, CSF2 and PTH, which are known to promote PCa development and progression (Figure 6b,d,g–i). We validated the expression of VEGF by immunoblotting to see the response of quercetin and resveratrol against PCa, and noticed a marked decrease especially in resveratrol and quercetin–resveratrol groups (Figure 6c). Vascular endothelial growth factor (VEGF) is a proangiogenic factor and a popular target to suppress angiogenesis by inhibiting its production [45]. Interestingly, VEGF is upregulated by epidermal growth factor receptor (EGFR), while on the other hand, VEGF upregulation independent of EGFR signaling contributes to resistance to EGFR inhibition [46]. EGFR overexpression is also observed frequently in circulating tumor cells (CTC) during PCa metastasis [47]. Expressions of VEGF and EGFR correlate with the metastatic characteristics, and therefore the inhibition of both has been shown to exert additive antitumor effects [45,46]. Our PCR array and its validation data (Figure 4a,b) clearly show a significant decrease in EGFR in all three treatment groups, suggesting the inhibitory role of quercetin and resveratrol against both, VEGF and EGFR signaling. EGFR expression was further confirmed using immunoblot, where we found marked inhibition at the protein level in response to resveratrol, quercetin and combination treatments (Figure 6c). 

Accumulating evidence suggests that intracellular calcium (Ca^2+^) homeostasis is altered in cancers, leading tumor development, progression and metastasis [48,49]. Therefore, targeting dysregulated Ca^2+^ signaling for cancer management has become a promising new area of research. The upstream analysis of quercetin–resveratrol modulated genes shows the inhibition of calcium signaling (Ca^2+^) (Figure 6d). However, our PCR array data show that androgen receptor (AR), which is one of the molecule involved upstream of Ca^2+^ signaling, was slightly higher and does not appear to support the upstream inhibition of Ca^2+^ signaling. As AR is specifically known to be regulated at the posttranslational level [50], we decided to validate AR expression at the protein level using immunoblotting. Although high variability was noticed in AR protein expression, a slight decrease was seen in treatment groups especially in resveratrol and quercetin–resveratrol groups (Figure 6e). The splicing factor heterogeneous nuclear ribonucleoprotein A1 (hnRNPA1) has been shown to play a major role in the alternative splicing of the AR [51], and has been shown to be a direct target of quercetin [52]. Thus, we determined the effect of our treatments on the protein expression of hnRNPA1. Our results show that quercetin and/or resveratrol treatments result in a decrease in hnRNPA1 levels, although it appears that the protein is reduced in both quercetin and resveratrol alone treatments, in addition to the combination (Figure 6f). This suggests that quercetin–resveratrol combination may be able to modulate AR signaling in PCa.

The PI3K upstream inhibition (Figure 6g) predicted based on quercetin–resveratrol modulated genes is in accordance to our previous study showing inhibition of PI3K/AKT pathway and BCL2 family of proteins in response to resveratrol treatment in PCa cells [12]. Colony-stimulating factor 2 (CSF2) was also predicted to be inhibited in this series (Figure 6h). This cytokine is thought to control the production and function of granulocytes and macrophages. In a genome-wide mRNA expression analysis in response to RUNX2 transcription factor inhibition, CSF2 induction in PCa cells was suggested to contribute to increased bone turnover in bone metastatic sites [53]. Next, the upstream analysis predicted inhibition of parathyroid hormone (PTH) signaling (Figure 6i). Interestingly, serum levels of PTH were shown to be positively correlated with prostate-specific antigen (PSA) in humans. Moreover, serum PTH and calcium each were found to be correlated with free PSA, further suggesting the importance of inhibition of PTH and Ca^2+^ signaling observed in this study.

An important observation noticed in these upstream analyses is the involvement of BCL2 and IL6 in all five signaling pathways shown in Figure 6b,d,g–i. Here, it is important to mention that upregulation of BCL2 is required for the progression of PCa from an androgen-dependent to an androgen-independent growth stage [54]. In our study, BCL2 appeared as a crucial molecule showing connections with several other molecules identified in this study. Similarly, Interleukin-6 (Il6) was found to be significantly inhibited in all three treatment groups (Figure 4a,b). IL6 is a pro-inflammatory cytokine that is expressed at elevated levels in PCa and correlates negatively with tumor survival and response to chemotherapy [55]. 

Next, NK3 homeobox-1 (NKX3.1), a homeobox-containing transcription factor, was found to be significantly upregulated in response to resveratrol, quercetin and combination treatments (Figure 4a,b). NKX3.1 also appeared in gene network 2 and in functional annotation of cumulative action of quercetin–resveratrol modulated genes (Figure 5b and Figure 6a). We validated the protein expression of NKX3.1 by immunoblotting and found marked increase in all three treated groups (Figure 6j). This is an important finding as NKX3.1 is a prostate-specific tumor suppressor gene and its loss predisposes mice and humans to PCa progression [56,57]. NKX3.1 is largely expressed in a prostate-specific and androgen-regulated manner. Loss of NKX3-1 protein expression is a common finding in human prostate carcinomas and prostatic intraepithelial neoplasia [56,58].

Additionally, the PCR array identified certain other genes, which were significantly modulated in response to quercetin–resveratrol treatment. For example, inhibition of early growth response 3 (EGR3) in quercetin and quercetin–resveratrol groups (Figure 4a,b). EGR3 is known to transcriptionally regulate genes involved in controlling the biological rhythm. EGR3 has been found to be overexpressed in non-relapsing PCa but not in relapsing PCa [59]. Similarly, the PCR array identified an increased level of APC (WNT signaling pathway regulator) (Figure 4a,b), which is a tumor suppressor protein antagonistic to the WNT signaling pathway [60]. Although the WNT pathway has been found to cause tumors and cancer development, APC genetic mutations have not been found to be a large component of PCa development [60]. Overall, our results suggest that a combination of quercetin and resveratrol treatments modulate several key genes and pathways known to be dysregulated in PCa, and therefore may be useful in PCa management.

## 3. Materials and Methods 

### 3.1. TRAMP Mice

All animal experiments were approved by the University of Wisconsin (UW) Institutional Animal Care and Use Committee and carried out in accordance with the National Institutes of Health Guide for the Care and Use of Laboratory Animals under IACUC Protocol #M01600. The TRansgenic Adenocarcinoma of Mouse Prostate (TRAMP) model (The Jackson Laboratory; C57BL/6-Tg(TRAMP)8247Ng/J (Stock # 003135)) closely resembles the development of human PCa. These mice spontaneously develop prostate tumors that mimic human PCa due to genetic modification to the promoter of rat probasin (rPB) encoding gene that targets expression of the SV40 large tumor T antigen. This results in the progression of PCa through prostatic intraepithelial neoplasia (PIN; precancerous lesions), well-differentiated carcinoma, poorly differentiated adenocarcinoma of the prostate gland, and finally metastasis to other areas of the body. The TRAMP mouse breeding colony was established and maintained by the UW Laboratory Animal Resources staff and genotyped in our lab to confirm proper genotype before study enrollment. Mice were allowed to acclimatize for one week prior to study initiation. Throughout the experiment, mice were housed with groupmates, up to 4 per cage, and monitored weekly for general health, body weight, and food consumption. During the study, a large number of TRAMP mice (age-matched) were not available at one given time to start all the treatment groups simultaneously. Therefore, we started the experiments with the available number of animals (spread evenly into the treatment groups) and followed by the addition of mice to each group as they became available until we obtained the required number (*n* = 12) in each group.

### 3.2. Experimental Design and Treatments 

Quercetin (>98% purity; Cayman Chemical, Ann Arbor, MI, USA) and/or Resveratrol (>98% purity; Sabinsa Corporation, NJ, USA) were fortified into AIN-76A diet by ENVIGO (Madison, WI, USA) at concentrations of 60 and 600 mg/kg diet, respectively. These doses are equivalent to 10 mg/kg b. wt. for quercetin and 100 mg/kg b. wt. for resveratrol. The rationale of these doses was extrapolated from multiple studies [13,14,20,22]. The control animals received AIN-76A diet and all animals received water ad libitum. Mice were separated into four experimental groups: (1) control (AIN76A diet), (2) quercetin (60 mg/kg in diet), (3) resveratrol (600 mg/kg in diet), and (4) quercetin (60 mg/kg in diet) + resveratrol (600 mg/kg in diet). Our plan for starting quercetin and resveratrol treatment and termination of the experiment is provided in Figure 1a. Mice were maintained until 28 weeks of age, at which point they were euthanized and tumor pictures, size and tumor wet weight were assessed. All samples were divided into two subsets that were either flash-frozen in liquid nitrogen before storage at −80 °C or formalin-fixed and stored for further experimentation.

### 3.3. Preparation of Tumor Protein Lysates and RNA

Flash-frozen tumors were pulverized using a pestle, cryovials, and liquid nitrogen. The ground powder was divided for protein and RNA analyses. For protein isolation, the powder was resuspended in RIPA lysis buffer (Millipore Sigma, Burlington, MA, USA) with freshly added PMSF (Amresco, Solon, OH, USA) and protease inhibitor cocktail (ThermoFisher Scientific, Waltham, MA, USA). The homogenate was incubated on ice for 30 min prior to centrifugation at 14,000× *g* for 30 min at 4 °C. The supernatant (tissue lysate) was collected and concentration determined by BCA Protein Assay (ThermoFisher Scientific, Waltham, MA, USA) per manufacturer’s protocol, then stored at −80 °C for further use. For RNA analysis, the powder was subjected to the manufacturer’s protocol using RNeasy Plus Mini Kit (Qiagen, Hilden, Germany) and quantified using a BioTek Synergy H1 (Winooski, VT, USA) Multimode plate reader.

### 3.4. Quantitative Reverse Transcription PCR (RT-qPCR) Analysis

For RT-qPCR analysis, equal amounts of RNA from three mice from each experimental group were pooled to make two separate groupings to represent averages of the entire cohort. RNA was transcribed using first-strand cDNA synthesis with random primers, dNTPs and M-MLV reverse transcriptase (Promega, Madison, WI, USA). RT-qPCR was performed using QuantStudio 3 (ThermoFisher Scientific) with SYBR Premix Ex Taq II (TaKaRa, Mountain View, CA, USA) with first-strand cDNA, forward and reverse primers (see Table S2). Genomic DNA Contamination Control Assay (Bio-Rad, Hercules, CA, USA #10025352) was used to ensure no genomic DNA contamination was present. Relative target mRNA was calculated using the ΔΔCT comparative method using GAPDH and β-actin as endogenous controls. The assay was performed using two biological pools in technical triplicate within each group.

### 3.5. Immunoblot Analysis

For immunoblot analysis, equal amounts of protein from two mice from each experimental group were pooled to make three separate groupings to represent averages of the entire cohort. Tissue lysates (15 μg total protein per well) were resolved using AnykD polyacrylamide gels (BioRad, Hercules, CA, USA). Proteins were transferred to 0.42 μm nitrocellulose membrane, then blocked with 5% non-fat dry milk in PBS-T. Membranes were probed with the appropriate primary antibody (see Table S3) and secondary horseradish peroxidase (HRP) conjugate antibody (Cell Signaling Technology, Danvers, MA, USA). Protein bands were detected by chemiluminescence using ECL Western Blotting Substrate or SuperSignal West Femto Chemiluminescent Substrates (Pierce ThermoFisher Scientific, Waltham, MA), using a Li-Cor Odyssey Fc (LI-COR, Lincoln, NE, USA). Full images of the immunoblots are shown in Figures S3–S5.

### 3.6. PCa PCR Array Analysis

To profile the expression of key PCa-related genes, RT^2^ Profile Mouse Prostate Cancer PCR arrays (Qiagen, Hilden, Germany; #PAHS-135Z) were performed per manufacturer’s protocol using SYBR Premix Ex Taq II (TaKaRa, #RR820). Resulting CT values were uploaded onto the data analysis portal provided by Qiagen (http://www.qiagen.com/us/shop/genes-and-pathways/data-analysis-center-overview-page/). Four reference genes (*Actb*, *Gapdh*, *Gusb* and *Hsp90ab1*) from the housekeeping gene (HKG) panel were used to normalize the data with high stringency and accuracy. Selected genes from the PCR array results (≥ 2-fold change in one group with statistical significance, and 1.5-fold change in any other group) were validated using RT-qPCR analysis. Relative target mRNA levels were calculated using the ΔΔCT comparative method and *Actb* and *Gapdh* endogenous controls. *p*-value < 0.05 calculated based on a Student’s t-test of the replicate 2^−ΔΔCT^ values for each gene in the control and treatment groups.

### 3.7. Ingenuity Pathway Analysis (IPA)

To understand the pathways modulated by quercetin and/or resveratrol, a list of differentially expressed genes from the PCR array (cut-off criteria ≥2-fold change in one group with statistical significance and ≥1.5-fold change in any other group) were compiled and analyzed using Qiagen’s IPA web portal (www.ingenuity.com). The predicted gene–gene interaction network, functional annotations and upstream regulators were generated using inputs of gene identifiers and fold-changes between control and treated group comparisons.

### 3.8. Immunohistochemical (IHC) Analysis

Formalin-fixed tissues were paraffin-embedded, sectioned, and mounted on serial slides at the University of Wisconsin Carbone Cancer Center (UWCCC) Experimental Animal Pathology Lab. One serial slide from each mouse was stained with hematoxylin and eosin (H&E). For IHC analysis, the slides were deparaffinized in xylenes and then rehydrated in an ethanol gradient from 100% to 60%. The slides were then washed in distilled water, steamed for 40 min in 1× IHC Epitope retrieval solution, and cooled for 20 min at room temperature. The slides were washed in TBS-T, and endogenous peroxidase activity was blocked by immersing the slides in 3% hydrogen peroxide for 10 min. The slides were washed, and non-specific binding was blocked via 60 min incubation in 5% blocking solution with normal goat serum in TBS-T. The slides were incubated with the appropriate primary antibody (see Table S3) overnight at 4 °C in a humidified chamber. Next day, slides were washed, incubated in secondary antibody (Vectastain ABC-AP Kit; Vector Lab, Burlingame, CA, USA) for 1 h, washed again, incubated in the ABC-HRP reagent for 30 min, washed a third time, subjected to Vector Red until appropriate staining intensity was observed, and washed in tap water. The nuclei in the tissue were counterstained with hematoxylin for 8 s, rinsed in tap water, and the slides were dehydrated via ethanol concentration gradient from 60% to 100% and two 5 min rounds of xylenes. The slides were then coverslipped using a 1:1 Permount:xylene mounting solution before drying and imaging. An EVOS XL Core Cell Imaging System (ThermoFisher Scientific, Waltham, MA, USA) was used to obtain multiple images of each tissue section at 40× magnification. The location of these images was based on the tumor tissue in section, presence of good contrast in magenta to blue staining, and uniform cell pattern. Semi-quantitation was performed using ImageJ Fiji (version 1.2) and statistical significance was determined using GraphPad Prism 5 software.

### 3.9. Statistical Analysis

Statistical analyses were plotted using GraphPad Prism 5 software (GraphPad Software, Inc., San Diego, CA, USA). The statistical test applied for each data are indicated in their respective figure legends. Data are expressed as mean ± SEM of three replicates, and statistical significance are denoted as * *p* < 0.05, ** *p* < 0.01, *** *p* < 0.001, compared to control.

## 4. Conclusions

Worldwide, PCa is the second most frequently diagnosed cancer and the fifth leading cause of cancer death in males [61]. PCa incidence increases with age, which makes it more difficult to manage this disease. Recent studies demonstrating the beneficial effect of modifying dietary habits have been suggested to manage multiple diseases, including PCa [3,62,63]. Here, we found that dietary supplementation with the grape antioxidants quercetin and resveratrol together had significant antitumor effects against PCa in both prevention and intervention settings. Additionally, the modulation of several key genes in response to quercetin–resveratrol treatment further supports the anti-PCa effects of the combination, including those involved in AR signaling, PI3K/AKT signaling, apoptosis, PTEN signaling, and the cell cycle. Our study also suggests that the stage of PCa development at which quercetin–resveratrol is administered may affect the outcome of the treatment. However, further investigations are necessary to substantiate these findings in human situations. Overall, these studies indicate that using a combination of quercetin and resveratrol may be a potential new treatment regimen for the prevention and/or treatment of prostate cancer and should be explored further.

## Figures and Tables

**Table 1:** Significantly altered genes in response to quercetin and/or resveratrol treatments in TRAMP mice. Fold change is relative to control tissues and all values are normalized to *Actb*, *Gapdh*, *Gusb*, and *Hsp90ab1* reference genes.

Gene	Quercetin		Resveratrol		QR		Cellular Location ^1^	Protein Type ^2^
	Fold Change	*p* Value	Fold Change	*p* Value	Fold Change	*p* Value		
*Apc*	1.05	0.2826	2.00	0.0000	1.54	0.0027	N	Enzyme
*Ar*	2.33	0.0073	1.19	0.2867	2.79	0.0013	N	NR
*Bcl2*	−2.19	0.0011	−1.60	0.0206	−1.19	0.4120	C	Transporter
*Cav2*	2.49	0.0569	1.27	0.0741	2.23	0.0009	PM	Other
*Dkk3*	−5.10	0.0010	−7.21	0.0008	−5.90	0.0009	ES	Cytokine
*Egfr*	−1.95	0.0678	−2.67	0.0075	−5.54	0.0021	PM	Kinase
*Egr3*	−4.82	0.0021	−1.25	0.4571	−1.85	0.0522	N	TR
*Etv1*	2.12	0.0005	1.94	0.0004	1.25	0.0358	N	TR
*Hal*	−2.01	0.0020	−1.68	0.0099	−1.11	0.2460	C	Enzyme
*Igf1*	−2.64	0.0017	−3.56	0.0008	−2.08	0.0039	ES	GR
*Igfbp5*	−1.23	0.4209	4.48	0.0013	−2.29	0.0048	ES	Other
*Il6*	−6.37	0.0014	−6.57	0.0012	−6.06	0.0023	ES	Cytokine
*Lgals4*	5.55	0.0010	1.71	0.0233	1.16	0.3417	ES	Other
*Loxl1*	−6.62	0.0060	−3.45	0.0289	−1.74	0.3113	ES	Enzyme
*Msx1*	−3.52	0.0003	−3.89	0.0002	−1.42	0.0167	N	TR
*Nkx3−1*	5.34	0.1512	5.01	0.0440	1.95	0.1645	N	TR
*Nrip1*	−4.04	0.1678	−8.78	0.0202	−9.63	0.0202	N	TR
*Ptgs1*	2.93	0.0128	2.44	0.0025	1.80	0.0517	C	Enzyme
*Sfrp1*	−4.68	0.0010	−11.30	0.0000	−3.12	0.0001	PM	TmR
*Slc5a8*	3.52	0.0540	−10.36	0.0019	1.13	0.6243	PM	Transporter
*Tfpi2*	−1.92	0.0003	−3.28	0.0001	−1.28	0.1904	ES	Other
*Tmprss2*	6.56	0.0435	1.94	0.0134	1.28	0.3619	PM	Peptidase

^1^ N = Nucleus, C = Cytoplasm, PM = Plasma Membrane, ES = Extracellular Space; ^2^ NR = nuclear receptor, TR = transcription regulator, GR = growth factor, TmR = transmembrane receptor.

## Supplementary Materials

The following are available online at https://www.mdpi.com/2072-6694/12/8/2141/s1, Figure S1: Tumor weight after removal of mice with no tumors at end of study, Figure S2: PCR array validation that resulted in inconclusive or non-significant outcomes, Figure S3: Full immunoblots from images used in Figure 3, Figure S4: Full immunoblots from images used in Figure 5, Figure S5: Full immunoblots from images used in Figure 6, Table S1: Results of RT^2^ Profile Mouse Prostate Cancer RT-qPCR array, Table S2: Primers used for RT-qPCR validation, Table S3: Antibodies used for immunhistochemistry and immunoblotting.
