# Supplementary material for: Quercetin–Resveratrol Combination for Prostate Cancer Management in TRAMP Mice
Source: Cancers (Basel). 2020 Aug 2;12(8):2141. doi: 10.3390/cancers12082141 (PMC7465013; doi:10.3390/cancers12082141)
Supplement: Supplementary file 1 [file cancers-12-02141-s001.pdf]

cancers

Supplementary Materials: Quercetin-Resveratrol Combination for Prostate Cancer Management in TRAMP mice

Chandra K. Singh, Gagan Chhabra, Mary A. Ndiaye, Imtiaz A. Siddiqui, Jennifer E. Panackal, Charlotte A. Mintie and Nihal Ahmad

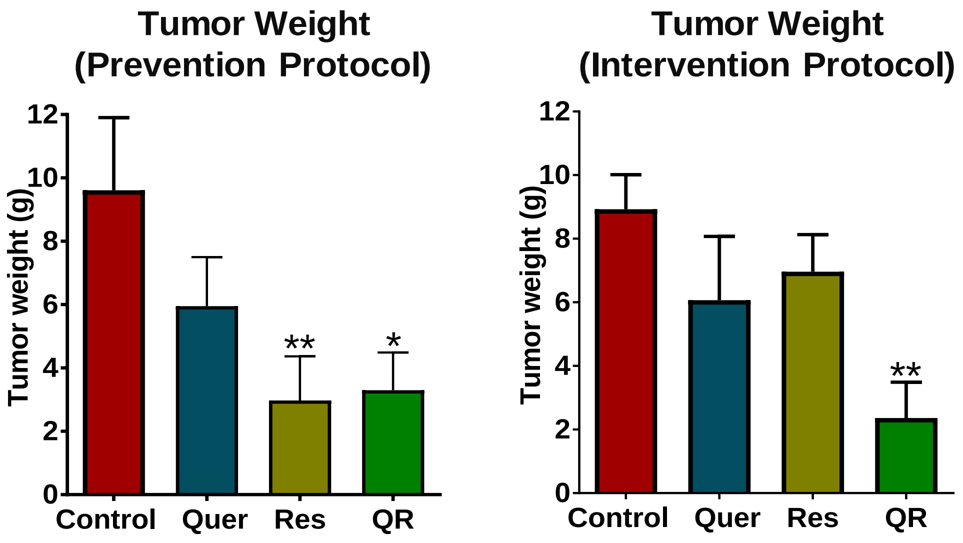

Figure S1. Tumor weight after removal of mice with no tumors at end of study. The re-analysis of tumor weight data presented in Figures 1C and 1E after the exclusion of TRAMP mice which failed to develop tumors (3 mice per group). Data is shown as mean ± SEM of 9 animals per group. GraphPad Prism 5 Software (GraphPad Software Inc.) was used to perform statistical analyses on tumor data using a one-way analysis of variance (ANOVA) followed by Sidak multiple comparison tests (*p < 0.05, **p < 0.01).

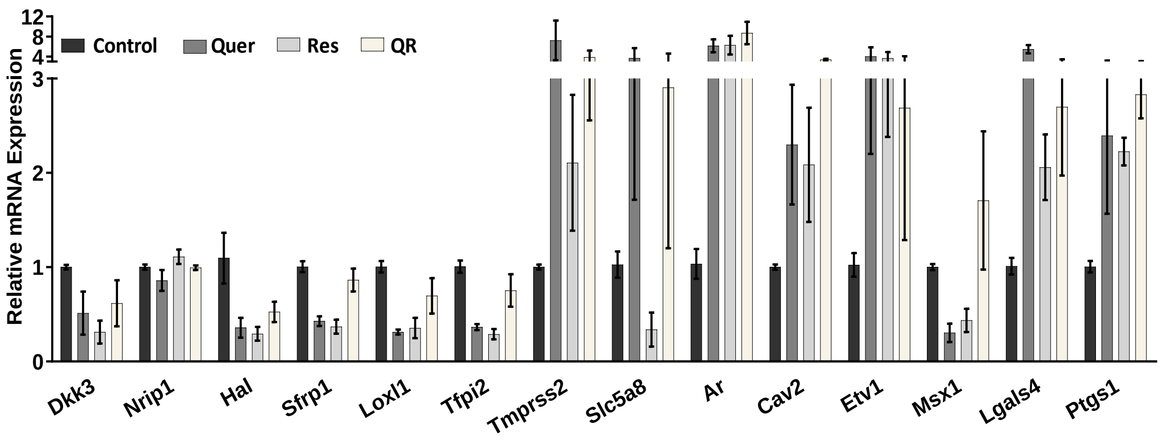

Figure S2. PCR array validation that resulted in inconclusive or non-significant outcomes. Data is presented as the mean ± SEM of two biological pools of three animals per group (n=6) in technical triplicate. A one-way ANOVA with Tukey's multiple comparison test was performed using GraphPad Prism 5 Software (**p < 0.01, *** p < 0.001, ****p < 0.0001).

Immunoblots from Figure 3

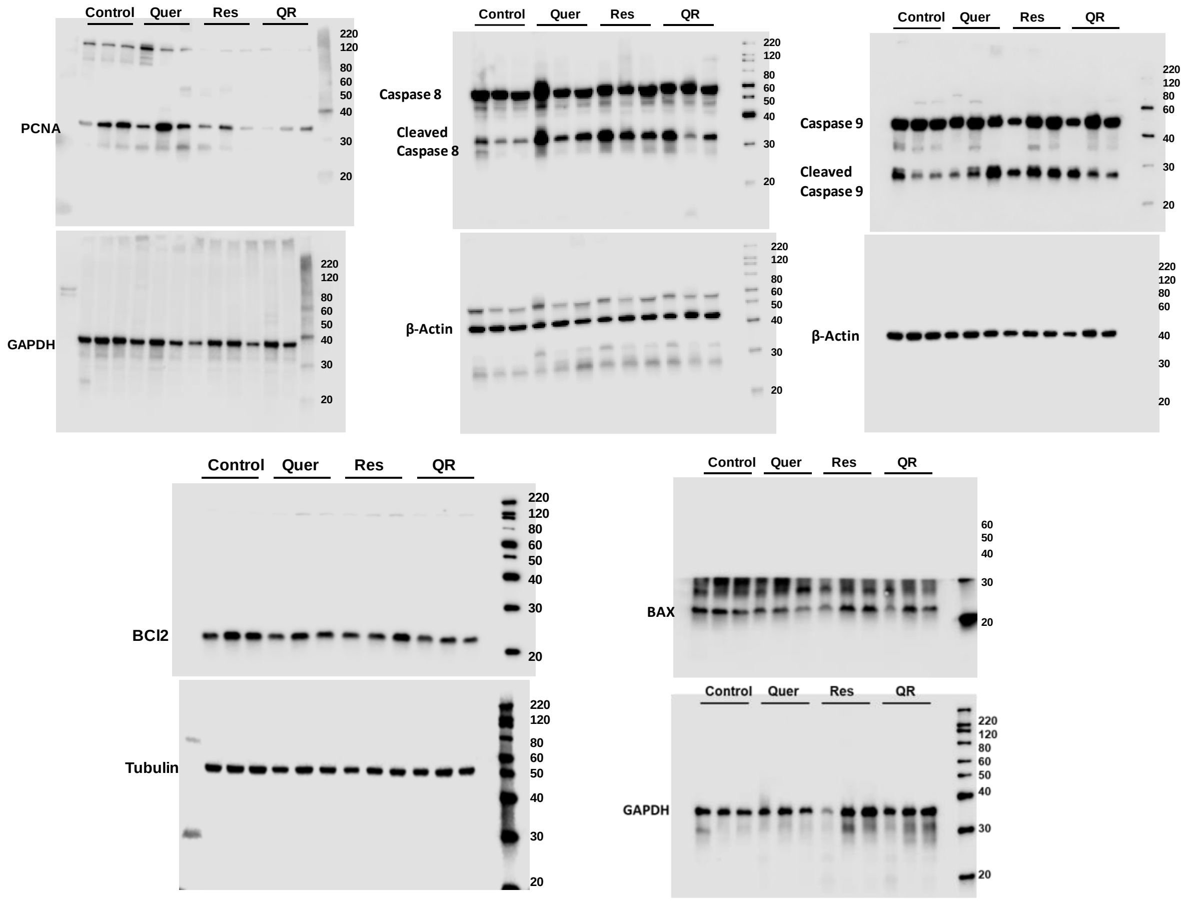

Figure S3. Full immunoblots from images used in Figure 3. After incubating with appropriate primary and secondary antibodies, blots were exposed to chemiluminescent developer substrate and images were acquired using a Li-Cor Odyssey Fc imager. Numbers on the right indicate molecular weight markers (kDa), and lanes are labeled on the top. Quer = quercetin; Res = resveratrol, QR = quercetin+resveratrol combination treatments.

Immunoblots from Figure 5

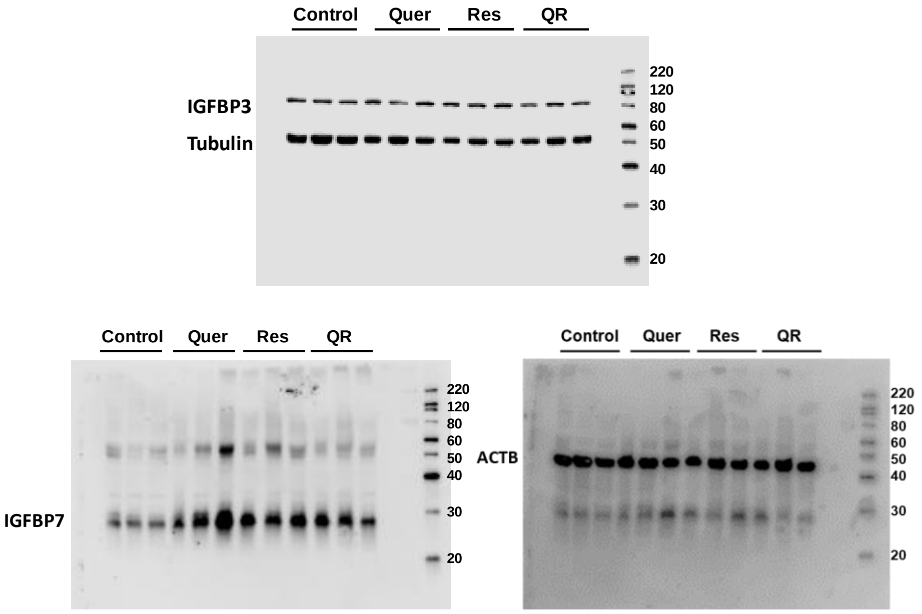

Figure S4. Full immunoblots from images used in Figure 5. After incubating with appropriate primary and secondary antibodies, blots were exposed to chemiluminescent developer substrate and images were acquired using a Li-Cor Odyssey Fc imager. Numbers on the right indicate molecular weight markers (kDa), and lanes are labeled on the top. IGFBP3 molecular weight appeared higher than the antibody company suggested, but we tried multiple antibodies and the same band arose, so we are confident that this is the correct band. Quer = quercetin; Res = resveratrol, QR = quercetin+resveratrol combination treatments.

Immunoblots from Figure 6

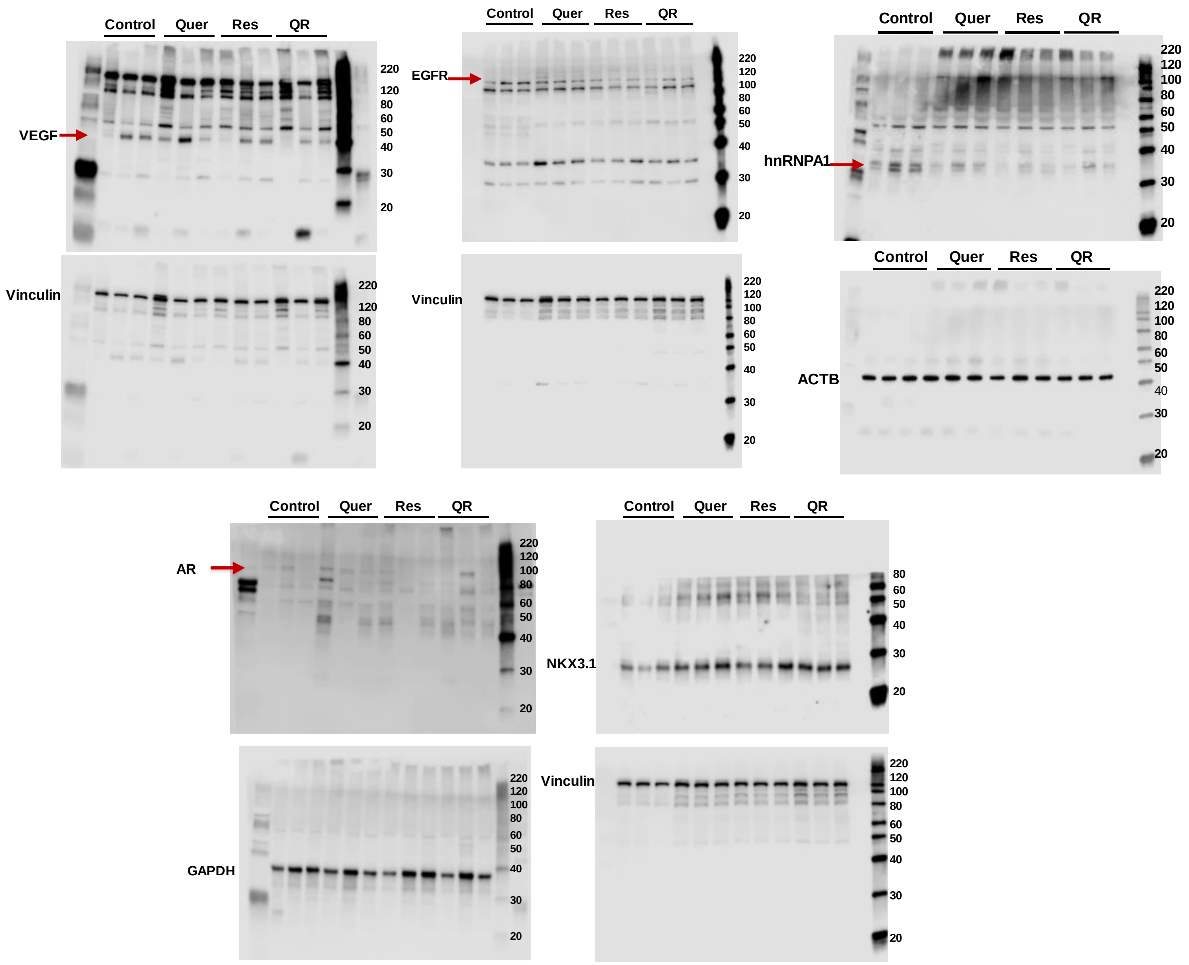

Figure S5. Full immunoblots from images used in Figure 6. After incubating with appropriate primary and secondary antibodies, blots were exposed to chemiluminescent developer substrate and images were acquired using a Li-Cor Odyssey Fc imager. Numbers on the right indicate molecular weight markers (kDa), and lanes are labeled on the top. Quer = quercetin; Res = resveratrol, QR = quercetin+resveratrol combination treatments.

Table S1. Results of RT² Profile Mouse Prostate Cancer RT-qPCR array.

RefSeq #	Gene	Description	Quercetin		Resveratrol		Quer+Res	
			Fold Change	p Value	Fold Change	p Value	Fold Change	p Value
**NM_011076**	***Abcb1a***	ATP Binding Cassette Subfamily B Member 1A	1.21	0.1381	−1.43	0.0001	1.15	0.2305
NM_011075	*Abcb1b*	ATP Binding Cassette Subfamily B Member 1B	−1.09	0.1941	1.58	0.0023	1.61	0.0216
NM_133360	*Acaca*	Acetyl-Coenzyme A carboxylase alpha	−1.01	0.8037	1.57	0.0004	1.16	0.0544
NM_009652	*Akt1*	Thymoma viral proto-oncogene 1	−1.03	0.6058	1.09	0.0331	−1.17	0.0221
NM_007462	*Apc*	Adenomatosis polyposis coli	1.05	0.2826	2	2E−05	1.54	0.0027
NM_0l3476	*Ar*	Androgen receptor	2.33	0.0073	1.19	0.2867	2.79	0.0013
NM_007489	*Arntl*	Aryl hydrocarbon receptor nuclear translocator−like	1.08	0.1236	1.54	0.0012	−1.07	0.122
NM_009741	*Bcl2*	B−cell leukemia/lymphoma 2	−2.19	0.0011	−1.6	0.0206	−1.19	0.412
NM_018883	*Camkk1*	Calmodulin−dependent protein kinase kinase 1α	1.07	0.2718	1.61	0.0004	1.41	0.0234
NM_009810	*Casp3*	Caspase 3	1.21	0.0241	1.05	0.3509	1.05	0.4354
NM_0l6900	*Cav2*	Caveolin 2	2.49	0.0569	1.27	0.0741	2.23	0.0009
NM_007628	*Ccna1*	Cyclin A1	1.5	0.0027	1.75	0.0082	1.06	0.6412
NM_007631	*Ccnd1*	Cyclin D1	1.75	0.1115	1.37	0.1402	−1.1	0.3761
NM_009829	*Ccnd2*	Cyclin D2	−1.2	0.2476	−1.01	0.9553	−1.02	0.8615
NM_009864	*Cdh1*	Cadherin 1	2	0.1774	−1.22	0.302	−2.9	0.0163
NM_007669	*Cdkn1a*	Cyclin−dependent kinase inhibitor 1A (P21)	−1.11	0.3973	−1.01	0.9997	−1.28	0.1547
NM_009877	*Cdkn2a*	Cyclin−dependent kinase inhibitor 2A	1.01	0.7876	1.17	0.003	−1	0.9669
NM_009907	*Cln3*	Ceroid−lipofuscinosis, neuronal 3	−1.12	0.2105	−1.12	0.0489	−1.59	0.0011
NM_l33828	*Creb1*	CAMP responsive element binding protein 1	−1.17	0.0898	1.31	0.0115	1.06	0.465
NM_001001602	*Dab2ip*	Disabled homolog 2 (Drosophila) interacting protein	−1.25	0.0776	1.32	0.0104	−1.11	0.2779
NM _007829	*Daxx*	Fas death domain−associated protein	1.29	0.0058	1.31	0.006	1.05	0.3468
NM_001003919	*Ddx11*	DEAD/H−Box Helicase 11	1.15	0.0449	1.34	0.0176	−1.06	0.5094
NM_0l5814	*Dkk3*	Dickkopf homolog 3 (Xenopus laevis)	−5.1	0.001	−7.21	0.0008	−5.9	0.0009
NM_015802	*Dlc1*	Deleted in liver cancer 1	1.22	0.1364	1.28	0.0281	1.53	0.009
NM_007900	*Ect2*	Ect2 oncogene	−1.12	0.1549	−1.09	0.4252	−1.11	0.2306
NM_007912	*Egfr*	Epidermal growth factor receptor	−1.95	0.0678	−2.67	0.0075	−5.54	0.0021
NM_0l8781	*Egr3*	Early growth response 3	−4.82	0.0021	−1.25	0.4571	−1.85	0.0522
NM_133659	*Erg*	Avian erythroblastosis virus E−26 (v−ets) oncogene	−1.48	1E−04	−1.87	0.0004	1.32	0.0548
NM_007960	*Etv1*	Ets variant gene 1	2.12	0.0005	1.94	0.0004	1.25	0.0358
NM_007988	*Fasn*	Fatty acid synthase	−1.07	0.213	1.52	8E−05	1.07	0.218
NM_0l9739	*Foxo1*	Forkhead box O1	1.26	0.0765	1.64	7E−05	−1.11	0.084
NM_007836	*Gadd45a*	Growth arrest and DNA−damage−inducible 45 alpha	−1.16	0.0716	2.13	7E−06	−1.22	0.1687
NM_145523	*Gca*	Grancalcin	−1.11	0.3079	1.07	0.3442	−1.01	0.9237
NM_008145	*Gnrh1*	Gonadotropin releasing hormone 1	1.46	0.0011	1.23	0.0212	1.18	0.1421
NM_008161	*Gpx3*	Glutathione peroxidase 3	1.36	0.0475	−1.23	0.199	−1.64	0.0046
NM_013541	*Gstp1*	Glutathione S−transferase, pi 1	1.2	0.0141	1.35	0.0005	−1.12	0.0597
NM _010401	*Hal*	Histidine ammonia lyase	−2.01	0.002	−1.68	0.0099	−1.11	0.246
NM_008255	*Hmgcr*	3−hydroxy−3−methylglutaryl−Coenzyme A reductase	1.11	0.0212	1.21	0.0116	1.12	0.0801
NM_010512	*Igf1*	Insulin−like growth factor 1	−2.64	0.0017	−3.56	0.0008	−2.08	0.0039

NM_010518	*Igfbp5*	Insulin−like growth factor binding protein 5	−1.23	0.4209	4.48	0.0013	−2.29	0.0048
NM_001314054	*Il6*	Interleukin 6	−6.37	0.0014	−6.57	0.0012	−6.06	0.0023
NM _0087 15	*Ints6*	Integrator complex subunit 6	−1.22	0.1195	1	0.9801	−1.69	0.0168
NM_026167	*Klhl13*	Kelch−like 13 (Drosophila)	1.17	0.1556	−1.38	0.0037	1.7	0.0014
NM_008455	*Klkb1*	Kallikrein B, plasma 1	3.29	0.1626	1.69	0.2935	1.2	0.5783
NM_010706	*Lgals4*	Lectin, galactose binding, soluble 4	5.55	0.001	1.71	0.0233	1.16	0.3417
NM_010729	*Loxl1*	Lysyl oxidase−like 1	−6.62	0.006	−3.45	0.0289	−1.74	0.3113
NM_011949	*Mapk1*	Mitogen−activated protein kinase 1	1.21	0.0129	1.65	0.0007	−1.15	0.1081
NM_008558	*Max*	MYC associated factor X	1.27	0.0073	1.03	0.3936	−1.14	0.226
NM_010835	*Msx1*	Homeobox, msh−like 1	−3.52	0.0003	−3.89	0.0002	−1.42	0.0167
NM_026658	*Mto1*	Mitochondrial translation optimization 1 homolog	1.22	0.0112	1.32	0.0092	1.03	0.5966
NM_013865	*Ndrg3*	N−myc downstream regulated gene 3	1.21	0.0441	1.38	0.0007	1.37	0.0171
NM_008689	*Nfkb1*	Nuclear factor kappa B subunit 1	−1.03	0.8932	−1.19	0.0479	−1.09	0.0765
NM_010921	*Nkx3.1*	NK3 homeobox 1	5.34	0.1512	5.01	0.044	1.95	0.1645
NM_173440	*Nrip1*	Nuclear receptor interacting protein 1	−4.04	0.1678	−8.78	0.0202	−9.63	0.0202
NM_011062	*Pdpk1*	3−phosphoinositide dependent protein kinase 1	1.2	0.035	1.61	6E−05	1.19	0.0308
NM_022889	*Pes1*	Pescadillo homolog 1, containing BRCT domain	−1.01	0.7537	1.1	0.0084	−1.17	0.0127
NM_001034085	*Ppp2r1b*	Protein phosphatase 2 scaffold subunit Abeta	−1.11	0.4009	−1.13	0.597	−1.34	0.0531
NM_031869	*Prkab1*	Protein kinase, AMP−activated, beta1 subunit	1.31	0.0489	1.32	0.0002	−1.17	0.0478
NM_008960	*Pten*	Phosphatase and tensin homolog	−1.16	0.3381	1.26	0.1942	−1.01	0.9257
NM_008969	*Ptgs1*	Prostoglondin−endoperoxide synthase 1	2.93	0.0128	2.44	0.0025	1.8	0.0517
NM_011243	*Rarb*	Retinoic acid receptor, beta	−1.47	0.0004	1.03	0.4313	1.04	0.6097
NM_0l9713	*Rassf1*	Ras association (RalGDS/AF−6) domain member 1	1.24	0.0064	1.2	0.018	1.04	0.5815
NM_009029	*Rb1*	Retinoblastoma 1	1.01	0.7525	1.6	0.0001	−1.02	0.7864
NM_133242	*Rbm39*	RNA binding motif protein 39	1.3	0.0036	1.34	0.0028	1.11	0.017
NM_011254	*Rbp1*	Retinal binding protein 1, cellular	1.28	0.0597	1.57	0.001	−1.08	0.6927
NM_028148	*Scaf11*	SR−related CTD−ossocioted factor 11	−1.01	0.9016	1.57	0.0001	1.21	0.0345
NM_009859	*Sept7*	Septin 7	−1.02	0.507	1.2	0.0009	−1.13	0.0468
NM_018754	*Sfn*	Stratifin	−1.33	0.3665	−1.27	0.1838	−1.35	0.2488
NM_013834	*Sfrp1*	Secreted frizzled−related protein 1	−4.68	0.001	−11.3	6E−06	−3.12	6E−05
NM_011367	*Shbg*	Sex hormone binding globulin	1.73	0.0539	1.44	0.1444	1.3	0.2118
NM_145423	*Slc5a8*	Solute carrier family 5 (iodide transporter), member 8	3.52	0.054	−10.36	0.0019	1.13	0.6243
NM_007707	*Socs3*	Suppressor of cytokine signaling 3	−1.58	0.0041	−1.3	0.0183	−1.43	0.039
NM_009238	*Sox4*	SRY−box containing gene 4	1.28	0.0597	1.57	0.001	−1.08	0.6927
NM_011480	*Srebf1*	Sterol regulatory element binding transcription factor 1	1.28	0.2504	1.32	0.1081	−1.61	0.0446
NM_011492	*Stk11*	Serine/threonine kinase 11	−1.55	0.0857	1.15	0.3334	−1.33	0.5375
NM_028150	*Supt7l*	Suppressor of Ty 7 (S. cerevisiae)−like	1.13	0.0516	1.54	6E−05	−1.17	0.0424
NM_009364	*Tfpi2*	Tissue factor pathway inhibitor 2	−1.92	0.0003	−3.28	1E−04	−1.28	0.1904
NM_009365	*Tgfb1i1*	Transforming growth factor beta 1 induced transcript 1	1.07	0.2525	1.53	0.0006	1.46	0.0025
NM_011594	*Timp2*	Tissue inhibitor of metolloproteinose 2	1.02	0.8327	1.05	0.49	−1.18	0.0193
NM_011595	*Timp3*	Tissue inhibitor of metalloproteinose 3	1.22	0.0144	−2.11	0.0003	−1.19	0.0369
NM_015775	*Tmprss2*	Tronsmem brone protease, serine 2	6.56	0.0435	1.94	0.0134	1.28	0.3619
NM_011640	*Trp53*	Tronsformotion related protein 53	−1.26	0.0089	−1.04	0.233	−1.42	0.0003

NM_013700	*Usp5*	Ubiquitin specific peptidase 5 (isopeptidose T)	1.07	0.1824	1.31	0.0055	1.2	0.0672
NM_009505	*Vegfa*	Vascular endothelial growth factor A	1.08	0.0586	1.18	0.1779	−1.33	0.0086

Table S2. Primers used for RT−qPCR validation.

Gene	Amplicon Size (bp)	Primer Orientation	Primer Sequence (5' –> 3')	Tm (°C)	Location	Primer Bank ID
Apc	225	F	TCTGGGGACAAAGGTGGAAAT	61	915–935	158966685c2
		R	CTGGCCCGAGCCTCTTTAC	62.1	1139–1121	
Ar	137	F	CAGGAGGTAATCTCCGAAGGC	61.7	577–597	118129906c2
		R	ACAGACACTGCTTTACACAACTC	60.7	713–691	
Bcl2	108	F	GAGAGCGTCAACAGGGAGATG	62.2	469–489	133893253c2
		R	CCAGCCTCCGTTATCCTGGA	62.9	576–557	
Cav2	140	F	TCTCATCTCAAGCTAGGCTTCG	61.3	139–160	118131204c2
		R	GCCAGAAATACGGTCAGGAACT	62	278–257	
Dkk3	148	F	CAGCTCTCAACTACCCTCAGG	61.3	107–127	31560475c1
		R	ACCTCAGAGGACGTTTTAGCA	60.8	254–234	
Egfr	136	F	CAATGTTCCCATCGCTGTCGT	63	652–672	90403617c3
		R	TGTCTTTGCATGTGGCCTCAT	62.3	787–767	
Egr3	80	F	TTGCCTGACAATCTGTACCCC	61.8	58–78	118130757c1
		R	TAATGGGCTACCGAGTCGCT	62.6	137–118	
Etv1	184	F	GGTCTGCTTGCAGTCAAGAG	60.7	179–198	253683440c2
		R	GGTTTCGGAGTATGAGCTGTGT	62	362–341	
Hal	223	F	AATACCGGGAGCCTGAAAAATAC	60.4	317–339	31981709c3
		R	GCAGGGATTACAGTTCTAGCAAA	60.3	539–517	
Igf1	142	F	GTGGATGCTCTTCAGTTCGTGTG	61	473–495	*Origene Gene*
		R	TCCAGTCTCCTCAGATCACAGC	60.6	615–594	*ID* 16000
Igfbp5	154	F	CCAAGCACACTCGCATTTCC	61.9	413–432	70909321c2
		R	CCTTGTTCGGATTCCTGTCTCAT	61.9	566–544	
Il6	131	F	CTGCAAGAGACTTCCATCCAG	60.1	14–34	13624310c1
		R	AGTGGTATAGACAGGTCTGTTGG	60.8	144–122	
Lgals4	76	F	TGGGTACAACCCTCCACAGAT	62.3	519–539	343478165c2
		R	ATATGGCACACGCGGATTGAA	62.5	594–574	
LoxI1	175	F	TGCCCGACAACTGGAGAGA	62.5	371–389	255759948c1
		R	TGCGGATAGGGGAACTGCT	62.7	545–527	
Msx1	102	F	GCACAAGACCAACCGCAAG	61.9	504–522	113199782c2
		R	CGCTCGGCAATAGACAGGT	61.8	605–587	
Nkx3.1	125	F	GACTGTGAACATAATCCAGGGG	60.1	316–337	146134368c2
		R	TGATGGCTGAACTTCCTCTCC	61.2	440–420	
Nrip1	176	F	AGACCAGAACTTTAACCTCTCGG	61.3	141–163	27734110a1
		R	CGATGGAATCAGACAGCCTCT	61.3	316–296	
Ptgs1	129	F	ATGAGTCGAAGGAGTCTCTCG	60.4	1–21	144227245c1
		R	GCACGGATAGTAACAACAGGGA	61.7	129–108	
Sfrp1	86	F	TACTGGCCCGAGATGCTCAA	62.9	448–467	227908833c1
		R	GAGGCTTCCGTGGTATTGGG	62.3	533–514	
Slc5a8	108	F	TTATGGGCGGTCGCAGTATG	62.1	137–156	110347554c2
		R	CAAAACGGTAGACCTCGGCA	62.1	244–225	
Tfpi2	203	F	CTCTGCCAACAGACTTGCG	61	241–259	142382435c2
		R	GGTTCACACAAGCCCTTACAT	60.2	443–423	
Tmprss2	111	F	ATGCTCCGAGGATTACAACGC	62.5	155–175	34328225c2
		R	CGAGGGCTAAACACAGCGATT	62.7	265–245	
Bax	137	F	AGACAGGGGCCTTTTTGCTAC	62.3	62–82	133778943c1
		R	AATTCGCCGGAGACACTCG	62.1	198–180	
Nrf2	153	F	TAGATGACCATGAGTCGCTTGC	62.1	389–410	76573877c2
		R	GCCAAACTTGCTCCATGTCC	61.6	541–522	
Keap1	104	F	TGCCCCTGTGGTCAAAGTG	62.1	58–68	7710044a1
		R	GGTTCGGTTACCGTCCTGC	62.7	153–135	
Actb	154	F	GGCTGTATTCCCCTCCATCG	61.8	84–103	6671509a1
		R	CCAGTTGGTAACAATGCCATGT	61.1	237–216	
Gapdh	95	F	AGGTCGGTGTGAACGGATTTG	62.6	08–.28	126012538c1
		R	GGGGTCGTTGATGGCAACA	62.6	102–84	

Table S3. Antibodies used for immunhistochemistry and immunoblotting.

Protein Name	Supplier	Catalog Number	WB Dilution	Molecular Weight (kDa)	IHC Dilution
Ki67	Cell Signaling	12202	–	–	1:500
PCNA	Invitrogen	PA5–27214	–	–	1:500
Survivin	Cell Signaling	2802	–	–	1:500
4–Hydroxynonenal	Abcam	ab46545	–	–	1:150
PCNA	Santa Cruz Biotec.	sc–56	1:500	36	–
Caspase 8	Cell Signaling	9746	1:1000	43, 18	–
Caspase 9	Cell Signaling	9505	1:1000	47, 35	–
BCL2	Santa Cruz Biotec.	sc–7382	1:500	26	–
BAX	Cell Signaling	2772	1:1000	20	–
IGFBP3	Santa Cruz Biotec.	sc–6003	1:500	40	–
IGFBP7	Santa Cruz Biotec.	sc–6064	1:2000	29	–
VEGF	Proteintech	19003–1–AP	1:500	46	–
EGFR	Proteintech	18986–1–AP	1:1000	134	–
AR	Proteintech	22089–1–AP	1:1000	99	–
NKX3.1	Proteintech	10365–1–AP	1:500	26	–
hnRNPA1	Cell Signaling	8443	1:1000	34, 40	–
GAPDH	Proteintech	10494–1–AP	1:2000	36	–
Vinculin	Cell Signaling	13901	1:1000	124	–
ACTB	Cell Signaling	3700	1:1000	45	–
TUBB	Cell Signaling	2128	1:1000	55	–

© 2020 by the authors. Licensee MDPI, Basel, Switzerland. This article is an open access article distributed under the terms and conditions of the Creative Commons Attribution (CC BY) license (http://creativecommons.org/licenses/by/4.0/).
